# Development and Validation of the Athlete Food Insecurity Scale (AFIS)

**DOI:** 10.3390/nu18081189

**Published:** 2026-04-10

**Authors:** Gonca Yıldırım, Önder Sünbül, Murat Baş, Özlem Çetiner

**Affiliations:** 1 Department of Nutrition and Dietetics, Graduate School of Health Sciences, Acibadem Mehmet Ali Aydınlar University, 34752 Istanbul, Türkiye; gncyildirim@gmail.com; 2 Department of Nutrition and Dietetics, Faculty of Health Sciences, Toros University, 33140 Mersin, Türkiye; 3 Department of Measurement and Evaluation in Education, Faculty of Education, Mersin University, 33110 Mersin, Türkiye; ondersunbul@mersin.edu.tr; 4 Department of Nutrition and Dietetics, Faculty of Health Sciences, Acibadem Mehmet Ali Aydınlar University, 34752 Istanbul, Türkiye; 5 Department of Nutrition and Dietetics, Faculty of Health Sciences, Atilim University, 06830 Ankara, Türkiye; ozlem.cetiner@atilim.edu.tr

**Keywords:** food access, athletes, sports nutrition, psychometrics

## Abstract

**Background/Objective:** Athletes’ dietary needs are influenced by the physiological demands of their sport, so the impacts of disrupted food access may vary from those experienced by the general population. This study aimed to develop and validate the Athlete Food Insecurity Scale (AFIS), a sport-specific tool designed to measure food insecurity in athletes. **Materials and Methods:** The study included 500 young adult athletes from 18 different sports disciplines. The sample was divided for exploratory factor analysis (*n* = 300) and confirmatory factor analysis (*n* = 200). Standard procedures for scale development were followed, including content validity assessment, construct validity testing, convergent validity analysis, and reliability evaluation. **Results:** The final 23-item scale demonstrated a four-factor structure including performance changes, coping strategies, basic nutritional needs, and physical access restraints. Factor loadings ranged from 0.344 to 0.956, item–total correlations from 0.513 to 0.781, and Cronbach’s alpha coefficients from 0.827 to 0.937. Confirmatory factor analysis supported the modified model with acceptable fit indices (χ^2^/df = 2.41, RMSEA = 0.080, TLI = 0.900, CFI = 0.910), and standardized factor loadings ranged from 0.53 to 0.89 (*p* < 0.05). Subscale scores differed significantly across Household Food Security Survey Module food security categories, supporting convergent validity. **Conclusions:** The AFIS demonstrates strong psychometric properties and may provide a sensitive tool for identifying and monitoring sport-specific food insecurity among athletes.

## 1. Introduction

Athletes are a subpopulation vulnerable to undernutrition, which can negatively affect both health and performance outcomes [1]. Unlike the general population, athletes have specific dietary requirements to meet energy demands, support training adaptations, enhance recovery, and regulate body composition [2,3]. When dietary intake does not adequately meet these demands, low energy availability and relative energy deficiency in sport may occur. Low energy availability is associated with impaired performance, limited training adaptation, and increased injury risk [4]. Athletes with insufficient energy intake may also experience reduced coordination and concentration, irritability, depressive symptoms, lower endurance performance, and increased susceptibility to infection, fatigue, chronic disease, and micronutrient deficiencies [5,6,7]. Despite these risks, a significant number of athletes experience nutritional deficiencies at some point during their careers [8,9].

Nutritional deficits may result from multiple factors, including unintentional undereating, inadequate nutrition knowledge, time constraints, restrictive dietary practices, or eating disorders [10]. In addition, food insecurity has been identified as a potential contributor to nutritional deficits and low energy availability in athletic populations [11,12]. The increased dietary and physical demands of performance, along with sports-specific expenses like limited time for employment and food preparation/purchase, equipment and travel costs, and lack of payment for sports make athletes vulnerable to food insecurity [13,14]. However, studies have shown that people experiencing food insecurity tend to participate less in physical activities [15,16]. When applied to the sports environment, disruptions, uncertainties, and anxieties related to access to food may influence non-exercise activity levels, sports performance, effort perception, and participation in training/competition for athletes [12,17].

Taken together, these considerations indicate that athletes have sport-specific nutritional demands and performance-related consequences when these demands are not met, highlighting the need to contextualize food insecurity within the athletic setting. However, most studies assessing food insecurity among athletes have used the 6- or 18-item USDA Household Food Security Survey Modules (HFSSM), or derived items originally developed for household-level or general adult populations. Instruments designed for the general population may not capture food access disruptions that manifest through sport-specific consequences. Also, prevalence studies further suggest that evaluating whether sport-related nutritional requirements necessary to sustain training and performance are met—alongside overall diet quality—may improve identification of athletes at higher risk [18].

Given these factors, a conceptual re-evaluation of food insecurity in athletes and the development of a context-specific measurement instrument would allow for a more accurate assessment of athletes’ exposure to food access challenges. This study therefore aims to: (i) examine the underlying dimensions of food insecurity in athletes, (ii) develop a screening instrument to assess sport-specific food insecurity, and (iii) assess the validity and reliability of the developed scale among young adult athletes.

## 2. Materials and Methods

### 2.1. Ethical Considerations

The research was conducted in accordance with the principles of the Declaration of Helsinki. The ethical appropriateness of the study was reviewed and approved by the Acibadem University and Acibadem Healthcare Institutions Medical Research Ethics Committee, with approval granted under decision number 2022-08/17.

### 2.2. Stage 1: Item Generation

To establish the conceptual framework, a three-step approach was used: (i) a comprehensive literature review, (ii) interviews with individuals from the target population, and (iii) consultations with subject-matter experts [19]. The literature search was conducted using PubMed, Google Scholar, and the Open Access Theses and Dissertations databases. Publications from the past 20 years were identified using combinations of the keywords food security, food insecurity, food availability, food access, or food utilization and athletes. Reference lists of included studies were manually screened, and citation tracking was performed during manuscript preparation to identify additional relevant studies. Experts in the field were also consulted to ensure comprehensive coverage. Studies reporting food access among athletes as a primary or secondary outcome were included.

Target group interviews were conducted to explore athletes’ experiences related to food access. A semi-structured interview guide was developed based on the literature review, previously published qualitative studies, and domains outlined in the Position of the American Dietetic Association on Food Insecurity in the United States, particularly sections related to food and nutrition security assessment [20,21,22]. The guide and interview procedure were reviewed by a researcher with expertise in qualitative methods. In-depth interviews were conducted via Google Meet and lasted approximately 20–30 min. Interviews continued until data saturation was reached, defined as the point at which no new themes emerged. Ten athletes participated. Example questions included: (1) “Can you afford to buy the foods and drinks necessary for your sport?” (2) “What challenges do you face when you cannot afford these foods and drinks?” (3) “How do you manage this situation?” (4) “Can you access the foods and drinks you need at training or competition venues?” and (5) “Does training intensity or travel affect your eating habits?” To complement athlete perspectives, four coaches (two fencing, one athletics, and one boxing coach) were also interviewed to capture potential issues athletes might not report. All interviews were audio-recorded and transcribed verbatim for content analysis.

Based on the literature review and qualitative findings, 33 preliminary items were generated to represent four dimensions of sport-specific food insecurity: performance changes, coping strategies, basic nutritional needs, and physical access restraints.

### 2.3. Stage 2: Validity and Reliability

#### 2.3.1. Content Validity

Content validity of the draft scale was evaluated through target population feedback followed by expert review. A mixed-method approach was adopted, incorporating quantitative (item impact score) and qualitative (cognitive interviewing) procedures to ensure item clarity and representativeness. Ten licensed athletes from different sports disciplines assessed item relevance and comprehensibility. Following scale administration, brief interviews were conducted with each participant to confirm their understanding of the instrument and identify any unfamiliar or confusing terms. Items that caused hesitation or difficulty were reviewed with respondents, and necessary revisions were made based on feedback [22,23,24,25]. Participants rated each of the 33 items on a 5-point Likert scale ranging from 1 (not important) to 5 (very important). Item impact scores were calculated using the formula: Impact Score = Frequency (%) × Importance. Items with impact scores ≥ 1.5 were retained. The Likert-type response format was also evaluated. Participants were instructed to read each item carefully and respond based on the frequency that most accurately reflected their actual experiences, rather than the frequency they believed should occur. Response options were defined as: 0 (never), 1 (rarely), 2 (sometimes), 3 (usually), and 4 (always). An expert panel of 11 individuals—academics in Nutrition and Dietetics, Sports Sciences, and measurement and evaluation, as well as a sports dietitian and licensed athletes—independently evaluated each item and provided suggestions for potential item additions. Content validity was assessed using the Content Validity Index (CVI), calculated as the number of experts rating the item as appropriate divided by (total number of experts/2 − 1). For 11 experts, items with CVI values above 0.59 were retained [25].

Following content validity evaluation, the initial 33-item pool was revised and expanded to a final 35-item version.

#### 2.3.2. Sampling and Participants Characteristics for Construct Validity and Reliability

The 35-item scale was administered to athletes aged 18–26 years who had held a sports license for at least five years and were actively involved in competitive sport. Data were collected between March and April 2023. A total of 500 athletes participated. The dataset was randomly divided into two independent subsamples: 300 participants (Sample 1) for exploratory factor analysis (EFA) and 200 participants (Sample 2) for confirmatory factor analysis (CFA) [26,27]. Participants were recruited using snowball sampling through in-person visits to universities and sports clubs, as well as online dissemination via social media platforms commonly used by athletes. At the beginning of the survey, participants were informed that participation was voluntary, that they could withdraw at any time, and that the data would be used solely for research purposes. Data were collected via self-report without obtaining personal identifiers. To enhance response quality, selected items in the online survey included text-entry boxes to help detect inattentive responding. The survey platform required completion of all items to prevent missing data.

#### 2.3.3. Assessment of Construct Validity and Reliability

The suitability of the study data for exploratory factor analysis was assessed using the Kaiser–Meyer–Olkin (KMO) measure (>0.60) and Bartlett’s test of sphericity (χ^2^, *p* < 0.05) [28]. Items with factor loadings below 0.32 and items loading on more than one factor with a loading difference of less than 0.10 were removed from the scale. Exploratory factor analysis using principal axis factoring (PAF) was conducted to determine the factor structure of the scale. To enhance factor interpretability, promax rotation was applied. The number of factors was initially examined using the Kaiser criterion (eigenvalues > 1) and further evaluated using parallel analysis [29].

Convergent validity was assessed in the total sample (*N* = 500) by examining whether subscale scores differed across food security categories defined by the Household Food Security Survey Module (HFSSM) short form [30]. Significant differences in AFIS subscale scores across HFSSM categories were interpreted as evidence of convergent validity. The HFSSM classifies food security into four categories: high, marginal, low, and very low food security. In the six-item short form, responses of “yes,” “often,” “sometimes,” “almost every month,” and “some months but not every month” are considered affirmative. Affirmative responses are summed to obtain a food security score. Raw scores of 0–1 indicate high or marginal food security (with 1 classified as marginal), scores of 2–4 indicate low food security, and scores of 5–6 indicate very low food security. For reporting purposes, scores of 0–1 were classified as food secure, whereas the combined categories of low and very low food security were classified as food insecure [31]. The Turkish validity and reliability of the HFSSM short form was previously established by Emiral et al. (2017) [32].

The hypothesized model derived from the EFA was subsequently tested using confirmatory factor analysis (CFA) in Sample 2 (*n* = 200). Model fit was evaluated using the root mean square error of approximation (RMSEA), the Tucker–Lewis index (TLI), and the comparative fit index (CFI). Overall model fit was interpreted in conjunction with multiple fit indices [33,34].

Finally, the reliability of the scale was assessed (in total Sample) through internal consistency analyses, which included Cronbach’s alpha and item–total correlation coefficients. Scales with Cronbach’s alpha values between 0.80 and 1.00 and item–total correlation coefficients greater than 0.30 were considered to demonstrate high reliability [28].

### 2.4. Statistical Analysis

Descriptive statistics are presented as mean ± standard deviation (SD) or median (first–third quartile) for continuous variables and as frequencies and percentages (%) for categorical variables. Exploratory and confirmatory factor analyses were conducted to assess the construct validity of the scale. Reliability was evaluated using Cronbach’s alpha (α) coefficients and item–total correlations. Group differences were analyzed using one-way analysis of variance (ANOVA), and analysis of covariance (ANCOVA) was performed in analyses requiring adjustment for covariates. In all analyses, a *p* value < 0.05 was considered statistically significant. Data were analyzed using the Statistical Package for Social Sciences (SPSS v.22) and Analysis of Moment Structure (AMOS v.21). Parallel analysis was conducted in RStudio version 4.4.1.

## 3. Results

### 3.1. Characteristics of the Study Sample

Of the 500 athletes included in the study, 29.4% (*n* = 147) completed the questionnaire online via Google Forms, while 70.6% (*n* = 353) completed it through face-to-face administration. The mean age of participants was 21.1 ± 2.97 years. Most participants were male (*n* = 422, 84.4%), had a normal body mass index (*n* = 402, 80.4%), and nearly half were university students (*n* = 228, 45.6%). The majority of athletes (*n* = 394, 78.8%) lived in the family home. Among participants who reported a monthly income (*n* = 360, 72.0%), the median income was 6000 (2000–10,000) Turkish lira, and 46.2% (*n* = 230) reported receiving financial support from their family. Additionally, 18.4% of athletes (*n* = 92) reported earning income from sport. Based on HFSSM scores, participants were classified into three food security levels: high or marginal food security (0–1 points), low food security (2–4 points), and very low food security (5–6 points). Accordingly, 49.0% of participants (*n* = 245) had high or marginal food security, 26.4% (*n* = 132) had low food security, and 24.6% (*n* = 123) had very low food security. For reporting purposes, participants with scores of 0–1 were classified as food secure, while those with low and very low food security were grouped and classified as food insecure. Table 1 presents the descriptive characteristics and HFSSM classifications of the subsamples used for exploratory and confirmatory factor analyses in detail. Sub samples did not significantly differ on age, sex, BMI, educational status, income, food insecurity status, earning money from sports and sports related characteristics.

Participants represented 18 distinct sports disciplines, with football constituting the largest subgroup in the sample (*n* = 373, 74.6%). By sport type, 78.8% of participants (*n* = 387) competed in team sports, 7.8% (*n* = 39) in endurance sports, 7.2% (*n* = 36) in strength sports, and 7.6% (*n* = 36) in weight-class sports. Participants had been licensed athletes for an average of 8.4 ± 3.25 years and had competed for 7.5 ± 3.60 years. Mean weekly training time was 8.9 ± 5.43 h. Table 2 presents information on athletes’ sporting background and sport disciplines; sport disciplines are grouped by sport type and reported as frequencies and percentages.

### 3.2. Factor Structure of the Athlete Food Insecurity Scale

For Sample 1, the data were suitable for factor analysis, with a KMO value of 0.932 and a significant Bartlett’s test of sphericity [χ^2^(253) = 4843.6, *p* < 0.001]. Exploratory factor analysis using PAF with promax rotation resulted in the removal of 12 items with factor loadings below 0.32 or cross-loading differences below 0.15. The final scale comprised 23 items loading on four factors with eigenvalues greater than 1, accounting for 61.4% of the total variance. Parallel analysis suggested a six-factor solution; however, the additional factors were not retained due to lack of interpretability and unstable item loadings. Therefore, a four-factor solution was retained.

Factor 1 (Performance Changes) included eight items evaluating whether current dietary practices meet athletic demands. Factor 2 (Coping Strategies) consisted of seven items assessing responses to situations of food absence or insufficiency. Factor 3 (Basic Nutritional Needs) comprised four items assessing access to the fundamental requirements of sports nutrition. Factor 4 (Physical Access Restraints) comprised four items assessing constraints in accessing safe food within the sports environment. Explained variance and eigenvalues for each factor are presented in Table 3.

**Figure 1:**
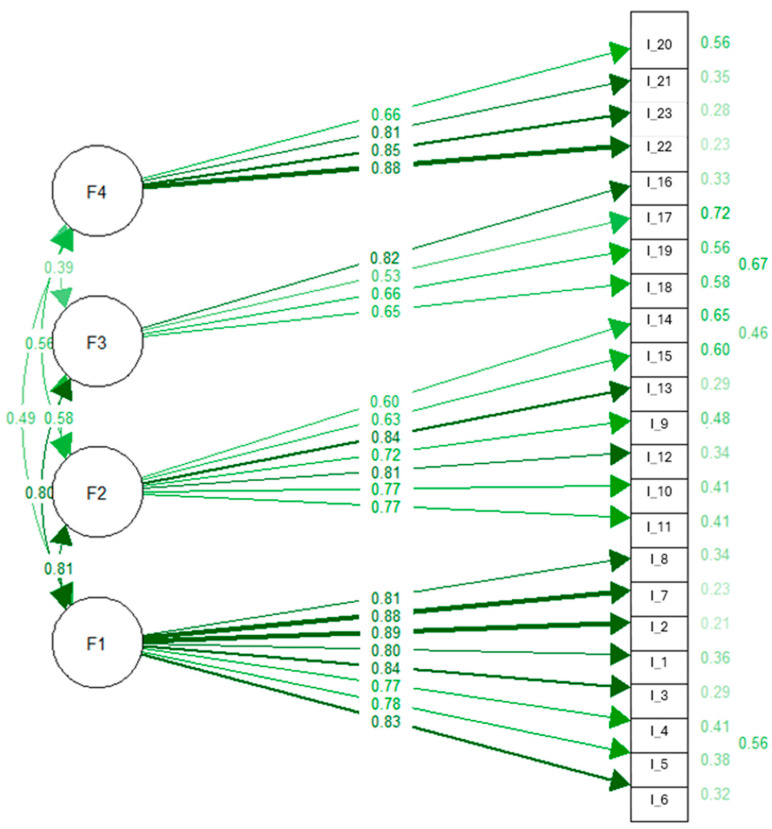
Confirmatory factor analysis model of the four-factor structure (M1). Darker lines indicate higher factor loadings.

In Sample 2, confirmatory factor analysis (CFA) was conducted on the 23-item scale obtained after the removal of 12 items based on the EFA results. All item–scale correlations exceeded 0.32. The initial four-factor model (M0) did not demonstrate adequate fit to the data (χ^2^/df = 3.05, *p* < 0.001, RMSEA = 0.101, TLI = 0.851, CFI = 0.868). Based on modification indices, error covariances were added between items 18–19, 14–15, and 4–5. The modified model (M1) showed improved fit compared to the initial model. As no further substantial modification indices were observed, M1 was accepted as a reasonable representation of the underlying factor structure. As presented in Table 4, the modified model demonstrated acceptable fit to the data (χ^2^/df = 2.41, *p* < 0.001, RMSEA = 0.080, TLI = 0.900, CFI = 0.910). The standardized factor loadings of the 23 items ranged from 0.53 to 0.89 (*p* < 0.05). The final CFA model is illustrated in Figure 1.

### 3.3. Convergent Validity

Across all subscales, AFIS scores increased as food security status worsened, and statistically significant differences were observed between the groups. These differences remained significant after controlling for monthly income. Post hoc analyses indicated that all pairwise group comparisons were significant for the performance changes, coping strategies, and basic nutritional needs subscales. For the physical access restriction subscale, the high/marginal food security group had significantly lower mean scores compared to the low and very low food security groups (Table 5).

### 3.4. Reliability

Cronbach’s alpha, an indicator of internal consistency, was 0.937 for the Performance Changes subscale, 0.884 for the Coping Strategies subscale, 0.827 for the Basic Nutritional Needs subscale and 0.853 for the Physical Access Restraints subscale. In the Sample 1, corrected item–total correlation coefficients ranged from 0.513 to 0.781 (Table 3).

### 3.5. Scoring and Interpretation of the AFIS

To reflect periods of active training and account for differences in competition schedules across sports, the reference period was defined as the previous 12 months. In sports with more frequent competition schedules, future studies may consider shorter recall periods to reduce potential recall bias. Likert-type response formats have been recommended for their robustness, and based on participant and expert feedback, the response options were retained as: 0 = never, 1 = rarely, 2 = sometimes, 3 = often, and 4 = always. Higher scores indicate greater severity in the corresponding dimension of food insecurity for each subscale. Since items 17, 18, and 19 are positively phrased, they were reverse-coded when calculating the Basic Nutritional Needs subscale score. The possible score ranges for the subscales are 0–32 for performance changes, 0–28 for coping strategies, 0–16 for basic nutritional needs, and 0–16 for physical access restraints. Intercorrelations among the four subscales ranged from 0.36 to 0.74, indicating moderate to strong associations between the dimensions. These findings suggest that the subscales represent related but distinct aspects of athlete food insecurity [27]. Importantly, these dimensions are conceptualized as facets of a single underlying construct rather than independent causal domains (e.g., economic, behavioral, or access-related determinants). Therefore, while a total score can be calculated to reflect overall food insecurity risk, it is recommended that subscale scores also be interpreted separately to capture the multidimensional nature of the construct.

## 4. Discussion

In this study, we developed the AFIS to identify athletes experiencing sport-specific consequences of disruptions in food access. A standard scale development approach was followed, combining qualitative methods—such as literature review, in-depth interviews with the target group, and expert consultation—with quantitative procedures including analyses of construct validity and reliability. The results supported a four-factor structure reflecting performance changes, coping strategies, basic nutritional needs, and physical access restraints, indicating that athlete food insecurity is a multidimensional construct.

### 4.1. Study Sample

Exploratory factor analysis was conducted in a sample of 300 young adult athletes, and confirmatory factor analysis was performed in a separate sample of 200 athletes. According to Comrey and Lee [35], a sample size of 300 is considered good for exploratory factor analysis. For confirmatory factor analysis, Kline [34] suggests that samples of around 200 participants are generally regarded as acceptable for obtaining stable and reliable model estimates.

As a result of the snowball sampling approach, the sample was predominantly composed of football players (74.6%), reflecting the distribution of licensed athletes in Türkiye. However, future studies should include a more balanced representation of weight-class, endurance, and strength sports. Although no items appeared to be sport- or culture-specific, the global generalizability of the scale should be evaluated in future research.

The higher representation of male participants in this sample aligns with the national distribution of licensed athletes in Türkiye (65.2% male, 34.8% female) [36]. However, female athletes may be more vulnerable to food insecurity due to gender-specific risk factors operating both within sport settings and in the general population. Women’s sports generally receive less financial support and media visibility than men’s sports, which may result in more limited income opportunities for female athletes [10]. In addition, young adult women may face broader social and economic disadvantages, including lower purchasing power and greater household or caregiving responsibilities, which may further increase their vulnerability to food insecurity [37]. Future screening and intervention studies should include more female athletes to better address their sport-specific nutritional and physiological needs, particularly in relation to menstrual function, bone health, and performance sustainability.

### 4.2. Factor Structure and Construct Representation

The findings support a four-factor structure reflecting the multidimensional nature of athlete food insecurity. The decision to retain four factors, despite parallel analysis suggesting a higher number, was based on theoretical interpretability, structural coherence, and the stability of the factor solution. Examination of the additional factors indicated that they were not conceptually meaningful and included items with weak or cross-loadings, limiting their interpretability. The identified dimensions—performance changes, coping strategies, basic nutritional needs, and physical access restraints—capture both traditional food insecurity experiences and sport-specific consequences. This structure is consistent with the conceptual framework developed during the qualitative phase of the study and suggests that food insecurity among athletes cannot be fully understood using general population measures alone. The factor loadings, the proportion of explained variance, and the internal consistency coefficients collectively indicate that the items adequately represent their respective dimensions and form a reliable measurement structure [34]. Overall, these results support the construct representation of the AFIS as a multidimensional tool designed to capture sport-specific experiences of food insecurity. Based on the CFA results, several items were retained despite modification suggestions, as they represent conceptually distinct aspects of athlete food insecurity. For example, items assessing reductions in training frequency and training duration were preserved because they reflect different dimensions of training disruption. A decrease in training frequency may indicate reduced opportunities to train, which can reflect more severe constraints on sport participation. In contrast, a reduction in training duration reflects a change in the intensity or volume of individual sessions and may occur for reasons such as fatigue, limited energy intake, or recovery problems [12,38]. These two situations have different implications for performance and athlete well-being. Moreover, qualitative interviews indicated that some athletes reported changes in one of these dimensions but not the other, supporting the decision to retain both items. Similarly, items related to body weight and body composition were retained because they capture different nutritional outcomes across sport types. In some sports, such as football, maintaining body weight may be sufficient, whereas in weight-class or physique-oriented sports, athletes may maintain weight while still being unable to achieve or sustain their desired body composition due to inadequate access to appropriate foods [39,40]. Therefore, both items were considered important for preserving the conceptual breadth of the construct across different sporting contexts. Two items reflecting coping strategies—needing recipe knowledge or skills to prepare cheaper sport-appropriate foods and bringing food to training or competition to reduce costs—were also retained. Although both relate to obtaining food at lower cost, they capture different aspects of coping [41]. One reflects individual knowledge, skills, and resourcefulness, while the other reflects behavioral adaptations to environmental or economic constraints. These distinctions are particularly relevant in contexts where access to affordable or appropriate foods is limited due to geographic, economic, or logistical barriers. Retaining both items therefore supports the content validity of the coping strategies subscale. Accordingly, conceptually relevant items with acceptable factor loadings were retained to preserve the multidimensional structure of the scale. The use of correlated residuals was limited and theory-driven, applied only to items with clear semantic overlap within the same subdimension; however, the stability of this structure should be further evaluated in independent samples.

Another evidence for construct validity was the expected pattern of significant differences in AFIS sub-scores across HFSSM food insecurity categories. However, HFSSM primarily identifies more severe manifestations of food insecurity, such as insufficient intake leading to weight loss. In the broader literature, severe food insecurity is associated with weight loss, whereas marginal food security may be linked to increased body weight through reliance on low-cost, energy-dense foods [42,43,44]. Accordingly, measurement instruments relying exclusively on these indicators may demonstrate greater sensitivity in detecting more severe stages of food insecurity. As AFIS reflects the identified sport-specific dimensions of food insecurity, it may offer improved construct coverage and facilitate the identification of context-specific experiences that may not be captured by existing instruments. Athletes, however, have sport-specific physiological demands that vary by age, sex, discipline, and competitive role. Inadequate or excessive energy intake may disrupt both body weight and body composition regulation, as well as performance outcomes [45]. For this reason, access to adequate sports nutrition should include not only sufficient food quantity, but also appropriate food quality, the ability to adjust intake according to training demands, and access to sports nutrition products when necessary. Studies have provided evidence that food-insecure individuals may be less physically active, and that food security is closely linked to the ability to obtain adequate food to sustain an “active life” [15,46]. Insufficient food intake has been associated with multiple adverse outcomes in athletes, including difficulty concentrating on performance-related tasks and declines in training performance [38]. In a qualitative study by Anziano examining food insecurity among collegiate athletes (*n* = 18), some participants reported fatigue and difficulties concentrating in academic settings; however, the majority perceived the impact on their athletic performance to be more substantial [21]. In this context, capturing performance-related consequences of disruptions in food access among athletes is particularly important. Moreover, qualitative evidence indicates that athletes experiencing food insufficiency develop various coping strategies. These include preparing their own meals, purchasing food in bulk, choosing lower-cost or lesser-known brands, buying only staple or non-perishable items, relying on cheaper and less nutritious options (e.g., fast food or snack foods), taking advantage of discounts, seeking free food, sleeping or drinking water to suppress hunger, stealing food, or consulting their coach for support [20,21]. Assessing the presence and frequency of such coping strategies may therefore help capture the deprivation-related and socially sensitive dimensions of the food insecurity experience. Finaly, intensive training schedules may contribute to meal skipping, and competitions held in locations with limited food options may result in interruptions in consistent access to healthy foods. Furthermore, training demands and the stress associated with competition can affect time management, which is closely linked to food accessibility, and may complicate budget and nutrition management—particularly among young athletes living away from their families [18,47]. Consistent with this perspective, the concept of access in this population was conceptualized not only in terms of food availability, but also in relation to economic conditions and housing circumstances that shape the food environment.

To date, only one instrument has been developed to assess food insecurity specifically among athletes. The Athlete–Sports Nutrition Access Questionnaire primarily focuses on institutional and environmental barriers to food access in collegiate settings [48]. In contrast, the AFIS was designed to capture sport-specific consequences of inadequate nutrition, including performance changes, coping strategies, and disruptions in food access. This broader construct coverage may allow the scale to identify experiences that are not fully captured by existing instruments. These factors may result in a greater proportion of athletes being classified within the at-risk spectrum of food insecurity. Given that nutrition-related challenges constitute a significant component of athletes’ overall well-being and performance capacity, screening food insecurity using a context-specific instrument may provide a more sensitive approach for evaluating newly enrolled athletes and monitoring changes over time among those who continue in sport.

### 4.3. Limitations and Future Directions

This study has several limitations that should be considered when interpreting the findings. First, although the sample size was sufficient for factor analytic procedures, the use of non-probability sampling may limit the representativeness and generalizability of the results. In addition, measurement invariance across sex, sport type, and competitive level was not examined, and therefore the consistency of the scale across different athlete subgroups remains to be established. Furthermore, temporal stability was not assessed, as test–retest reliability was not conducted.

An important limitation relates to the conceptual scope of the instrument. The AFIS does not differentiate between underlying causal mechanisms of food insecurity (e.g., economic constraints, behavioral adaptations, or access-related barriers). Instead, it captures how these mechanisms are experienced and manifested within the athletic context. Accordingly, the subscales capture distinct yet interrelated dimensions of athlete food insecurity; while they can be interpreted separately to reflect its multidimensional nature, they should not be considered independent determinants. This limits the ability to directly attribute observed patterns to specific causal pathways.

In line with this, the AFIS is primarily designed as a screening tool to identify athletes at risk of food insecurity, rather than as an instrument for determining specific intervention targets. Although it provides a multidimensional assessment of food insecurity experiences, it does not allow precise identification of the underlying drivers required for tailored intervention planning.

Additionally, the CFA model included theoretically justified correlated residuals, which may have improved model fit but could also affect model generalizability. Therefore, the factor structure should be further validated in independent samples.

Future research should examine measurement invariance across diverse athletic populations using multi-group confirmatory factor analysis and evaluate test–retest reliability to establish temporal stability. Longitudinal designs are also needed to assess sensitivity to change and to explore associations between AFIS scores and relevant outcomes such as performance, injury risk, and health indicators. Establishing empirically derived cut-off points may further enhance the scale’s utility for screening purposes. Finally, shorter versions of the instrument could be developed to facilitate its use in large-scale surveillance and applied sport settings.

## 5. Conclusions

The findings of this study indicate that the AFIS provides a comprehensive assessment of athletes’ experiences related to food insecurity and demonstrates a valid and reliable factor structure. The scale appears to be a useful tool for evaluating food insecurity in sport-specific contexts and may support both research and practical applications.

The observed results suggest that sport-specific food insecurity is an important construct that warrants further investigation, particularly in relation to athletic performance and sport participation. Future research may benefit from using the AFIS to better understand these associations across different athlete populations.

Based on the evidence, the development of targeted strategies to improve food access among athletes, such as nutrition education programs and food assistance initiatives, may be considered. Overall, the AFIS has the potential to contribute to research, practice, and policy discussions aimed at addressing food insecurity in athletic populations.

## Figures and Tables

**Table 1:** Demographic characteristics and HFSSM classification of athletes.

	Full Sample (*n*:500)	EFA Sample (*n*:300)	DFA Sample (*n*:200)
Characteristic			
Age (year) ^1^	21.1 ± 2.97 (18–26)	21.2 ± 2.99	21.0 ± 2.97
Sex ^2^			
Female	78 (15.6)	27 (9.0)	51 (25.5)
Male	422 (84.4)	273 (91.0)	149 (75.5)
BMI (kg/m^2^) ^1^	22.5 ± 2.79 (16.3–34.1)	22.4 ± 2.56	22.7 ± 3.12
Educational status ^2^			
High school graduate	194 (38.8)	129 (45.1)	65 (32.8)
University student	228 (45.6)	119 (41.6)	109 (55.1)
University graduate	62 (12.4)	38 (13.3)	24 (12.1)
Living arrangement ^2^			
Family home	394 (78.8)	245 (81.7)	149 (74.5)
Own home	21 (4.2)	13 (4.3)	8 (4.0)
Club facility	35 (7.0)	23 (7.7)	12 (6.0)
Student house	22 (4.4)	7 (2.3)	15 (7.5)
Student dormitory	28 (5.6)	12 (4.0)	16 (8.0)
Income (TRY/Year) ^3^	6000 (2000–10,000)	7000 (2000–10,000)	6000 (2000–10,000)
Source of income ^2^			
Full-time paid employment	109 (21.9)	63 (21.0)	46 (23.0)
Part-time paid employment	70 (14.1)	40 (13.3)	30 (15.0)
Student scholarship	43 (8.6)	24 (8.0)	19 (9.5)
Student loan	23 (4.6)	13 (4.3)	10 (5.0)
Athletic scholarship	59 (11.8)	38 (12.7)	21 (10.5)
Athlete salary	25 (5.0)	19 (6.3)	6 (3.0)
Match bonuses	8 (1.6)	7 (2.3)	1 (0.5)
Financial support from family	230 (46.2)	130 (43.3)	100 (50.0)
Food security status			
Food secure	245 (49.0)	152 (50.7)	93 (46.5)
High or marginal food security	245 (49.0)	152 (50.7)	93 (46.5)
Food insecure	255 (51.0)	148 (49.3)	107 (33.5)
Low food security	132 (26.4)	78 (26.0)	54 (27.0)
Very low food security	123 (24.6)	70 (23.3)	53 (26.5)

^1^ Mean ± standard deviation (minimum–maximum). ^2^ Frequency (percentage). ^3^ Median (first quartile–third quartile). Percentages are based on valid responses.

**Table 2:** Sport-related characteristics.

	Full Sample (*n*:500)	EFA Sample (*n*:300)	DFA Sample (*n*:200)
Sport Discipline	*n* (%)		
Team sports	387 (78.8)	252 (84.0)	135 (67.5)
Football	373 (74.6)	250 (83.3)	123 (61.5)
Basketball	14 (2.8)	2 (0.7)	12 (6.0)
Endurance sports	39 (7.8)	16 (5.3)	23 (11.5)
Middle-distance running	8 (1.6)	5 (1.7)	3 (1.5)
Long-distance running	8 (1.6)	3 (1.0)	5 (2.5)
Tennis	22 (4.4)	7 (2.3)	15 (7.5)
Race walking	1 (0.2)	1 (0.3)	-
Strength/power sports	36 (7.2)	17 (5.7)	19 (9.5)
Javelin throw	9 (1.8)	1 (0.3)	8 (4.0)
Hammer throw	3 (0.6)	2 (0.7)	1 (0.5)
Discus throw	1 (0.2)	0 (0.0)	1 (0.5)
Shot put	7 (1.4)	6 (2.0)	1 (0.5)
Sprint	8 (1.6)	4 (1.3)	4 (2.0)
Long jump	3 (0.6)	2 (0.7)	1 (0.5)
Triple jump	3 (0.6)	1 (0.3)	2 (1.0)
High jump	2 (0.4)	1 (0.3)	1 (0.5)
Combat sports	38 (7.6)	15 (5.0)	23 (11.5)
Boxing	4 (0.8)	3 (1.0)	1 (0.5)
Wrestling	9 (1.8)	1 (0.3)	8 (4.0)
Kickboxing	9 (1.8)	4 (1.3)	5 (2.5)
Taekwondo	16 (3.2)	7 (2.3)	9 (4.5)
Variable	Mean ± SD (min–max)		
Duration of licensed sports participation (years)	8.41 ± 3.25 (5–18)	8.56 ± 3.30 (5–18)	8.18 ± 3.17 (5–18)
Duration of competition participation (years)	7.49 ± 3.60 (1–18)	7.77 ± 3.52 (1–18)	7.08 ± 3.69 (1–18)
Weekly training duration (hours/week)	8.90 ± 5.43 (2–30)	8.96 ± 5.56 (2–30)	8.82 ± 5.24 (2–26)

**Table 3:** Item Loadings, Item–Total Correlations, and Variance Explained for the Four-Factor Model.

Sub Scales	Item No		Items	Factor 1	Factor 2	Factor 3	Factor 4	Item Total Correlation r	Variance Explained (%)
	First	Final							
**During the last 12 months, how often did you experience the following situations?**									
**For items 1–8, read each statement as completing the sentence.** *Because I could not afford adequate nutrition for my sport*									
**Performance Changes**	11	1	I felt more vulnerable to injury or illness.	0.657				0.675	45.2
	12	2	I had slower recovery after training or competition.	0.651				0.746	
	13	3	I had discomfort during or after training or competition (e.g., fatigue, cramps, pain).	0.675				0.733	
	14	4	I reduced the frequency of my training sessions.	0.926				0.736	
	15	5	I shortened the duration of my training sessions.	0.922				0.726	
	16	6	I reduced the intensity of my training sessions.	0.951				0.714	
	17	7	I had difficulty maintaining focus during training.	0.668				0.781	
	19	8	I reduced my physical activity outside of sport.	0.610				0.745	
**Coping Strategies**	22	9	I hid foods I ate from my coach or teammates because they were low-cost.		0.636			0.600	7.2
	23	10	I borrowed money from my coach or teammates for food.		0.905			0.632	
	24	11	I asked a teammate to share food because I could not afford it.		0.911			0.624	
	25	12	I took food from the training or competition venue because I could not afford it.		0.687			0.677	
	26	13	I ate less at other times of the day to save food for training.		0.516			0.706	
	28	14	I needed recipes or cooking skills to prepare sports foods at a lower cost.		0.373			0.636	
	29	15	I brought food to training or competitions to reduce costs.		0.421			0.642	
**Basic Nutritional Needs**	3	16	I was unsure whether I could afford the nutrition needed for my performance.			0.448		0.697	5.0
	4	17	I could afford sports nutrition products when needed.			0.582		0.580	
	9	18	I could afford the nutrition needed to maintain my ideal body weight.			0.956		0.529	
	10	19	I could afford the nutrition needed to maintain my ideal body composition.			0.903		0.587	
**Physical Access Restraints**	32	20	I skipped meals during long competition-related travel.				0.344	0.559	4.0
	33	21	I consumed unhygienic food at training or competition venues.				0.869	0.549	
	34	22	I consumed non-fresh food at training or competition venues.				0.917	0.513	
	35	23	I consumed foods with unreliable additives at training or competition venues.				0.853	0.555	
**Eigenvalues**				10.7	1.9	1.5	1.3	Total variance explained (%) 61.4	

**Table 4:** Goodness-of-Fit Indices for the Initial and Modified CFA Models.

Model	χ^2^/df	RMSEA	TLI	CFI
Initial model (M0)	3.05	0.101	0.851	0.868
Modified model (M1)	2.41	0.08	0.900	0.910

RMSEA = root mean square error of approximation; TLI = Tucker–Lewis index; CFI = comparative fit index.

**Table 5:** AFIS Subscale Mean Scores Across HFSSM Food Security Categories.

Subscales	High or Marginal (*n* = 245)	Low (*n* = 132)	Very Low (*n* = 123)	ANOVA F Value ^2^
Performance Changes ^1^	3.1 ± 4.76 ^a^ (0–32)	8.8 ± 6.61 ^b^ (0–27)	14.3 ± 6.43 ^c^ (0–31)	164.003 *
Coping Strategies ^1^	2.7 ± 4.1 ^a^ (0–24)	6.8 ± 5.03 ^b^ (0–18)	11.4 ± 5.36 ^c^ (0–23)	141.133 *
Basic Nutritional Needs ^1^	3.5 ± 3.02 ^a^ (0–13)	7.3 ± 3.38 ^b^ (0–15)	8.9 ± 3.38 ^c^ (0–16)	133.098 *
Physical Access Restraints ^1^	2.0 ± 2.89 ^a^ (0–16)	4.2 ± 3.37 ^b^ (0–13)	5.1 ± 3.36 ^b^ (0–16)	45.250 *

^1^ Mean ± standard deviation (minimum–maximum values) ^2^ One-way ANOVA was used; Tamhane post hoc tests were applied. ^a^^b^^c^ Groups sharing the same letter do not differ significantly. * *p* < 0.001.

## Data Availability

The data presented in this study are available on request from the corresponding author due to privacy and ethical restrictions.

## Abbreviations

The following abbreviations are used in this manuscript:

AFIS	Athlete Food Insecurity Scale
ANOVA	Analysis of Variance
ANCOVA	Analysis of Covariance
BMI	Body Mass Index
CFA	Confirmatory Factor Analysis
CFI	Comparative Fit Index
CVI	Content Validity Index
EFA	Exploratory Factor Analysis
HFSSM	Household Food Security Survey Module
KMO	Kaiser–Meyer–Olkin
PAF	Principal Axis Factoring
RMSEA	Root Mean Square Error of Approximation
TLI	Tucker–Lewis Index
